# Cardiac Rehabilitation in India: Results from the International Council of Cardiovascular Prevention and Rehabilitation’s Global Audit of Cardiac Rehabilitation

**DOI:** 10.5334/gh.783

**Published:** 2020-04-03

**Authors:** Abraham Samuel Babu, Karam Turk-Adawi, Marta Supervia, Francisco Lopez Jimenez, Aashish Contractor, Sherry L. Grace

**Affiliations:** 1 Department of Physiotherapy, Manipal College of Health Professions, Manipal Academy of Higher Education, Manipal, Karnataka, IN; 2 QU Health Department of Public Health, Qatar University, Doha, QA; 3 Gregorio Marañon University Hospital, Madrid, ES; 4 Division of Preventive Cardiology, Mayo Clinic, Rochester, Minnesota, US; 5 Centre for Rehabilitation Medicine and Sports Medicine, Sir H. N. Reliance Foundation Hospital and Research Centre, Mumbai, IN; 6 School of Kinesiology and Health Science, York University, Toronto, Ontario, CA; 7 KITE & Peter Munk Cardiac Centre, University Health Network, University of Toronto, Ontario, CA

**Keywords:** Cardiac rehabilitation, Cardiovascular disease, Barriers, India, physiotherapy

## Abstract

**Background:**

Cardiac rehabilitation (CR) is recommended in clinical practice guidelines for comprehensive secondary prevention. While India has a high burden of cardiovascular diseases (CVD), availability and nature of services delivered there is unknown. In this study, we undertook secondary analysis of the Indian data from the global CR audit and survey, conducted by the International Council of Cardiovascular Prevention and Rehabilitation (ICCPR).

**Methods:**

In this cross-sectional study, an online survey was administered to CR programs, identified in India by CR champions and through snowball sampling. CR density was computed using Global Burden of Disease study ischemic heart disease (IHD) incidence estimates.

**Results:**

Twenty-three centres were identified, of which 18 (78.3%) responded, from 3 southern states. There was only one spot for every 360 IHD patients/year, with 3,304,474 more CR spaces needed each year. Most programs accepted guideline-indicated patients, and most of these patients paid out-of-pocket for services. Programs were delivered by a multidisciplinary team, including physicians, physiotherapists, among others. Programs were very comprehensive. Apart from exercise training, which was offered across all centers, some centers also offered yoga therapy. Top barriers to delivery were lack of patient referral and financial resources.

**Conclusions:**

Of all countries in ICCPR’s global audit, the greatest need for CR exists in India, particularly in the North. Programs must be financially supported by government, and healthcare providers trained to deliver it to increase capacity. Where CR did exist, it was generally delivered in accordance with guideline recommendations. Tobacco cessation interventions should be universally offered.

## Introduction

India has a high burden of cardiovascular disease (CVD) [1], which, given the health system, results in high costs incurred to patients [2], with many of them being unable to afford even the basic preventive medications [3]. Thus, there is need for cost-effective measures for controlling CVD. Cardiac rehabilitation (CR) is one such cost-effective intervention [4].

CR is a well-established multidisciplinary model of care based evidence-based core components, such as structured exercise training and risk factor management [5]. Expert reviews [6,7]. and meta-analyses have established that participation in CR is associated with significant reductions in cardiovascular mortality, re-hospitalization [8]. as well as significant improvements in quality of life [9]. Benefits of CR among various groups are also demonstrated in India [10,11,12]. Indeed, based on the evidence, CR is a recommendation in clinical guidelines for CVD and heart failure [13], including in India [14,15].

Despite these benefits, CR remains grossly under-utilized on a global scale [16,17]. CR began in the West in the 1960s and has grown consistently since. In India, however, the development of CR has been slow. A narrative review several years ago highlighted several small studies from across the country, with most delivering in-hospital CR and only a few offering supervised out-patient phase-2 CR [18]. There has been no survey of CR programs in India nor quantification of CR need to our knowledge. To fill this gap, Indian data from the first-ever International Council of Cardiovascular Prevention and Rehabilitation (IC-CPR) global audit and survey on CR is summarized.

## Material and Methods

ICCPR, a member of the World Heart Federation, facilitated program identification for this audit. This was a cross-sectional study, details of which have been reported elsewhere [19,20]. In this report, we summarize CR availability and provision in India specifically. With regard to the former, CR density (i.e. number of CR spots per incident ischemic heart disease [IHD] case annually) was computed using Global Burden of Disease study estimates for annual IHD prevalence [21], juxtaposed against national CR capacity (i.e., median number of patients a program could serve per year multiplied by number of programs).

For countries which offered CR, respective cardiology and CR societies were contacted to identify and survey the programs. Given that there were no specific CR societies in India, champions in CR were enlisted. Programs meeting the following criteria were sought through a snowball sampling method: offering Phase 2 CR including an initial assessment, structured exercise, and at least one other strategy to control risk factors for CVD.

The programs identified were contacted via email with a link to the piloted survey [22], which assessed capacity and services. The survey was administered through REDCap, with data collection occurring from June 2016 to July 2017. All responders provided informed consent through an online form. If there was no response, two e-mail reminders were sent, two weeks apart.

Data were analysed using SPSS version 24. All initiated surveys were included. However, the number of responses for each question varied due to skip logic and missing data. Descriptive analyses were used to report these findings.

## Results

### Availability, Capacity, Density and Unmet Need

**Figure 1:**
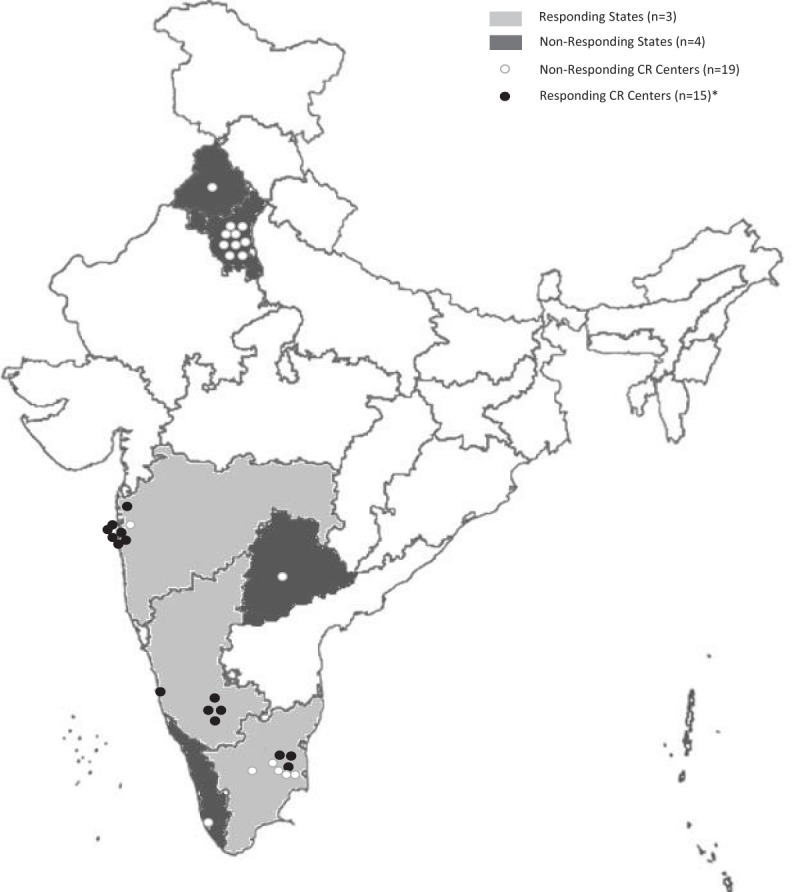
Distribution of cardiac rehabilitation centers in India. States not in grayscale did not have CR centers. * A total of 18 responses were received. However, three centers did not reveal their location.

Overall, 23 programs were identified across India (Figure 1), with programs in each of the following six Indian states and one Union territory identified: Kerala (n = 1), Karnataka (n = 4), Tamil Nadu (n = 8), An-dhra Pradesh (n = 1), Maharashtra (n = 8), Punjab (n = 1) and Delhi (n = 10).

Eighteen programs responded (response rate 78.3%), however, only 14 had completeness of data. The respondents were from the three southern states of India (Karnataka [n = 5; 100.0%], Maharashtra [n = 7; 87.5%] and Tamil Nadu [n = 3; 37.5%]; Figure 1). They reported serving a median of 200 patients/yr, but having a capacity to serve 400, such that 9200 patients could be served per year [18]. Given the 2016-estimate of 3,313,674 incident cases of IHD in India in each year, this suggests there is only one CR “spot” for every 360 patients in need [23]. This was among the lowest densities of any country with CR (61st of 86 countries where this could be computed). Overall, there is a need for 3,304,474 more CR ‘spots’ each year to treat IHD patients, without considering other indications such as heart failure. This is the greatest unmet need of any low and middle-income country globally (China comes a close second).

### Nature of CR Services in India

The earliest program began in 1997, with the most recent opening in 2014. Characteristics of these pro-grams are shown in Table 1, with elements delivered in Table 2 (note where education sessions were offered, patients were offered on average 4 session, of approximately 25 minutes duration; see supplemental table 2 elsewhere) [24]. Most commonly-accepted indications (see supplemental table 2 elsewhere) [19], most common healthcare professionals on CR teams (supplemental table 3 elsewhere) [19]; cardiopulmonary resuscitation training are shown in Supplemental Table 5 elsewhere [19]; we note two programs had yoga therapists on staff) and core components delivered (Supplemental Table 6 elsewhere) [19] are also shown by WHO region and in all countries within South-East Asia elsewhere [19], for comparison purposes. In that global paper, the high-quality of CR in India where it does exist is established (Supplementary Table 9 elsewhere) [19].

**Table 1:** Description of cardiac rehabilitation programs.

Nature of Program	n (%) or mean ± SD

CR setting	
Urban	10 (66.6%)
Suburban	5 (33.3%)
Rural	0 (0.0%)
Location of the CR program	
Tertiary care hospital	13 (92.8%)
Community hospital	0 (0.0%)
Rehabilitation hospital	1 (7.1%)
Not in hospital	3 (21.4%)
Program cost payment source*	
Patient	14/15; 93.3%
Insurance	4/15; 26.6%
Hospital	1/15; 6.6%
Government	1/15; 6.6%
Average direct cost to the patient where they pay (in Indian Rupees; n = 12)	₹5893.3 ± 3689.6 Median (IQR): ₹6000 (1500, 96000)
Referral frequency from Cardiology	
Regularly	5/14; 35.7%
Sometimes	9/14; 64.2%
Rarely	0
Cardiac indications for referral accepted by programs	
1. Post-MI/ACS	13/13 (100%)
2. Stable CAD, without a recent event or procedure	9/13 (69.2%)
3. Post PCI	10/13 (76.9%)
4. Post CABG	13/13 (100%)
5. Heart failure	11/13 (84.6%)
6. Patients who have had valve surgery/repair or TAVI	7/13 (53.8%)
7. Heart transplant	5/13 (38.5%)
8. Patients with ventricular assist devices	4/13 (30.8%)
9. Arrhythmias (hemodynamically-stable)	9/13 (69.2%)
10. Patients with implanted devices for rhythm control (i.e., ICD/CRT, pacemaker)	8/13 (61.5%)
11. Congenital heart disease	5/13 (38.5%)
12. Cardiomyopathy	7/13 (53.8%)
13. Rheumatic heart disease	6/13 (46.1%)
14. Pulmonary hypertension	1/13 (7.7%)
Non-cardiac indications for referral accepted by programs	11/13 (84.6%)
14, Patients at high-risk of cardiovascular disease (primary prevention)	2/13 (15.4%) 8/13 (61.5%)
Health care professionals on the CR team Cardiologist (n = 15)	
Dedicated to CR	3 (20%)
Part-time	10 (66.6%)
None	2 (13.3%)
Physiatrist (n = 15)	
Dedicated to CR	2 (13.3%)
Part-time	1 (6.6%)
None	12 (80%)
Sports Medicine Physician (n = 15)	
Dedicated to CR	0
Part-time	3 (20%)
None	12 (80%)
Other Physician (n = 15)	
Dedicated to CR	2 (13.3%)
Part-time	6 (40%)
None	7 (46.6%)
Physiotherapist (n = 15)	
Dedicated to CR	10 (66.6%)
Part-time	4 (26.6%)
None	1 (6.6%)
Nurse (n = 15)	
Dedicated to CR	3 (20%)
Part-time	5 (33.3%)
None	7 (46.6%)
Nurse practitioner (n = 15)	
Dedicated to CR	1 (6.6%)
Part-time	1 (6.6%)
None	13 (86.6%)
Psychiatrist (n = 14)	
Dedicated to CR	0
Part-time	5 (35.7%)
None	9 (64.2%)
Psychologist (n = 15)	
Dedicated to CR	1 (6.6%)
Part-time	9 (60%)
None	5 (33.3%)
Social worker (n = 15)	
Dedicated to CR	1 (6.6%)
Part-time	1 (6.6%)
None	13 (86.6%)
Dietitian (n = 15)	
Dedicated to CR	5 (33.3%)
Part-time	10 (66.6%)
None	0
Kinesiologist (n = 15)	
Dedicated to CR	1 (6.6%)
Part-time	2 (13.3%)
None	12 (80%)
Pharmacist (n = 15)	
Dedicated to CR	1 (6.6%)
Part-time	0
None	14 (93.3%)
Exercise specialist (n = 15)	
Dedicated to CR	5 (33.3%)
Part-time	1 (6.6%)
None	9 (60%)
Community health worker (n = 14)	
Dedicated to CR	1 (7.1%)
Part-time	1 (7.1%)
None	12 (85.7%)

* Respondents directed to select all that apply. **Abbreviations:** ACS – Acute coronary syndrome, CABG – Coronary artery bypass graft surgery, CR – Cardiac rehabilitation, CRT – Cardiac resynchronization therapy, ICD – Implantable cardioverter defibrillator, MI – Myocardial infarction, TAVI – Transcatheter aortic valve implantation, SD – standard deviation.

**Table 2:** Services delivered in cardiac rehabilitation centers across India (N = 15).

Element	n (%)

Initial assessment	15 (100.0%)
Individual consultation with a physician	14 (93.3%)
Individual consultation with a nurse	2 (13.3%)
Exercise stress test	12 (80.0%)
Other functional capacity test	Yes: 15 (100%)
Assessment of strength (e.g. handgrip)	Yes: 10 (66.6%)
Assessment of comorbidities/issues that could impact exercise (e.g. cognition, vision, musculoskeletal/mobility issues, frailty, and/or balance/fall risk)	Yes: 15 (100%)
Exercise prescription	Yes: 15 (100%)
Physical activity counseling	Yes: 15 (100%)
Supervised exercise training	Yes: 15 (100%)
Heart rate measurement training for patients	Yes: 15 (100%)
Resistance training	Yes: 15 (100%)
Management of cardiovascular risk factors	Yes: 15 (100%)
Prescription and/or titration of secondary prevention medications	Yes: 14 (93.3%)
Nutrition counseling	Yes: 15 (100%)
Depression screening	Yes: 12 (80%)
Psychological counseling	Yes: 13 (86.6%)
Smoking cessation sessions/classes	Yes: 11 (73.3%)
Vocational counseling/support for return-to-work	Yes: 10 (66.6%)
Stress management/relaxation techniques	Yes: 15 (100%)
Alternative forms of exercise, such as yoga, dance or tai-chi	Yes: 10 (66.6%)
Women-only classes	Yes: 2 (13.3%)
End of program re-assessment	Yes: 14 (93.3%)
Communication of patient assessment results with their primary care provider	Yes: 14 (93.3%)
Follow-up after outpatient program	Yes: 13 (86.6%)

Thirteen (72.2%) programs offered supervised home-based CR, two of which (11.1%) served 55% of their patients. No programs offered community-based CR; and only one (5.6%) program reported alternative models were reimbursed (See supplemental Table 1 elsewhere) [24]. Finally, researched rated perceived barriers to delivery, and programs in India most strongly endorsing lack of patient referral followed by financial resources [20].

## Discussion

Almost half of countries in the world do not have CR. Despite the availability of CR in India (given the high burden of CVD), the unmet need for CR is highest in India of any country in the world [19,20,23]. The programs that are available were clustered in the southern states of India, leaving major gaps in services in the North, East and West.

Where CR did exist, it was delivered in accordance with internationally-agreed guidelines [5]. Most programs accepted all cardiac indications as per clinical guidelines, and also accepted primary prevention and other chronic disease patients. Programs were delivered by a multidisciplinary team, including physicians (dedicated or consulting, which is likely appropriate). Physiotherapists were key, but also nurses, dietitians and mental healthcare providers were well-represented, such that all secondary prevention recommendations could be expertly delivered. Indeed, the programs were very comprehensive, although given the high degree of tobacco use in India [2,25], cessation interventions should likely be universally offered. Alternative forms of exercise were routinely offered including yoga, which is culturally relevant, and shown to be effective in a recent large randomized trial [26].

As previously documented [23,24], obtaining referrals to CR are one of the major challenges in India [27,28]. Physicians are likely not referring due to the dearth of programs (although they are not operating at capacity), and patient inability to pay for programs that do exist. In all but one responding program did patients not have to pay out-of-pocket; given that average annual income is ₹88,920 with a daily earning power around 247 (range: ₹138–₹1052) [29]; clearly CR is not affordable to patients. Once these system issues are addressed, electronic referrals could be instituted [30], and development of homebased models [17].

Caution is warranted in interpreting these results, with limitations for the global study elucidated elsewhere [19,20]. Given the sampling method, lack of a CR association and registry in India, there may be ascertainment bias. Response rate was good, but whether programs remain unidentified cannot be ruled out. However, even if a handful of programs were missed, clearly the conclusions regarding capacity would not be greatly affected. Furthermore, results are only generalizable to responding states. Second, the survey, while piloted, was not validated against actual delivery; knowing the CR guidelines, programs may have responded in a socially desirable manner, such that quality of CR delivery is not as high as reported.

Overall, it is clear that various strategies need to be implemented to improve CR delivery in India. Overcoming barriers at the health-care system, healthcare professionals and patient levels are vital to achieve this [28]. Increasing the number of CR centers along with policy for reimbursement of CR are needed. Methods to promote CR through local philanthropists, professional bodies and legislation are crucial to successful advocacy [31]. Physiotherapists appear to play a vital role in the delivery of CR in India. Therefore, a joint taskforce involving physiotherapists and cardiologists working towards improving CR in India is highly warranted to achieve these aims.

Capacity-building is a final key area to consider. The three CR training programs for healthcare professionals available globally are presented in the online Supplement. ICCPR offers the only certification program specific to low-resource settings; indeed approximately 1,000 physicians in India completed this training in 2018. Hopefully these physicians will go on to develop programs, as well as promote their trainees and collaborating allied healthcare professionals to also complete the certification, which will further enable CR development across India.

## Conclusions

The number and capacity of CR centers in India are grossly insufficient to meet the demands of the population with CVD. When compared to the rest of the world, India ranks poorly, even among low and middle-income countries. Yet, where it does exist, CR is of excellent quality, comprising a multi-disciplinary team, delivering very comprehensive services. Patients are almost universally paying for services out-of-pocket, and thus advocacy for reimbursement should be the priority for action, as it would also likely facilitate greater program proliferation.

## Additonal File

The additional file for this article can be found as follows:

10.5334/gh.783.s1 Supplemental Table 1. Summary of cardiac rehabilitation certifications for healthcare professionals.

## Abbreviations

₹ – Indian Rupee

CR – Cardiac rehabilitation

CVD – Cardiovascular disease

ICCPR – International Council of Cardiovascular Prevention and Rehabilitation

IHD – Ischemic heart disease

WHO – World Health Organization
