# Supplementary material for: Cardiac Rehabilitation in India: Results from the International Council of Cardiovascular Prevention and Rehabilitation’s Global Audit of Cardiac Rehabilitation
Source: Glob Heart. 2020 Apr 3;15(1):28. doi: 10.5334/gh.783 (PMC7218762; doi:10.5334/gh.783)
Supplement: Supplemental Table 1. — Summary of cardiac rehabilitation certifications for healthcare professionals. [file gh-15-1-783-s1.pdf]

Supplemental content:

Table 1: Summary of cardiac rehabilitation certifications for healthcare professionals

| Organisation (website) | Training Designation | Mode of delivery | Focus on low-resource setting |
|---|---|---|---|
| AACVPR (https://www.aacvpr.org/Certification/AACVPR-Professional-Certification) | Certified Cardiac Rehabilitation Professional | Off-site | No |
| BACPR (https://www.bacpr.com/pages/page_box_contents.asp?pageid=851&navcatid=182) | Exercise Instructor Qualification | On-site | No |
| ICCPR http://globalcardiacrehab.com/training-opportunities/certification/ | Cardiac Rehabilitation Foundations Certification | Online | Yes |

Abbreviations: AACVPR – American Association of Cardiovascular and Pulmonary Rehabilitation; BACPR – British Association of Cardiovascular Prevention and Rehabilitation; ICCPR – International Council for Cardiovascular Prevention and Rehabilitation
